# Retraction: Survivin knockdown alleviates pathological hydrostatic pressure-induced bladder smooth muscle cell dysfunction and BOO-induced bladder remodeling via autophagy

**DOI:** 10.3389/fcell.2023.1260130

**Published:** 2023-08-18

**Authors:** Frontiers Editorial Office

Following publication, concerns were raised regarding the validity of the data in the article. Following provision of raw data by the authors, the Chief Editor concluded that the article’s conclusions and assertions were not sufficiently supported by the findings from the material provided; therefore, the article has been retracted.

This retraction was approved by the Chief Editor of Frontiers in Cellular and Developmental Biology and the Editor-in-Chief of Frontiers. The authors did not agree to this retraction.

